# Prediction of emergency cerclage outcomes in women with cervical insufficiency: The role of inflammatory, angiogenic, and extracellular matrix-related proteins in amniotic fluid

**DOI:** 10.1371/journal.pone.0268291

**Published:** 2022-05-10

**Authors:** Kyong-No Lee, Kyo Hoon Park, Yu Mi Kim, Iseop Cho, Tae Eun Kim

**Affiliations:** Department of Obstetrics and Gynecology, Seoul National University College of Medicine, Seoul National University Bundang Hospital, Seongnam, Korea; University Hospital Bern, SWITZERLAND

## Abstract

**Objective:**

We aimed to determine whether various novel inflammatory, angiogenic, and extracellular matrix-related mediators in amniotic fluid (AF) can independently predict emergency cerclage outcomes in women with acute cervical insufficiency (CI).

**Methods:**

This was a retrospective cohort study conducted among 50 singleton pregnant women (18–25 weeks) who underwent emergency cerclage for CI and were subjected to amniocentesis. The AF samples were assayed for endoglin, endostatin, haptoglobin, insulin-like growth factor-binding protein (IGFBP)-3, -4, kallistatin, lumican, macrophage colony-stimulating factor (M-CSF), pentraxin 3, p-selectin, receptor for advanced glycation end products (RAGE), resistin, transforming growth factor beta-induced (TGFBI), and vitamin D-binding protein (VDBP) levels. Interleukin (IL)-6 levels in the AF were also measured for comparison with potential biomarkers assessed in this study. The primary endpoint was spontaneous preterm delivery (SPTD) at <34 weeks following emergency cerclage.

**Results:**

The AF levels of pentraxin 3, RAGE, and resistin were significantly higher in women who had SPTD at <34 weeks after cerclage placement (pentraxin-3: *P* = 0.003; RAGE: *P* = 0.041; and resistin; *P* = 0.002). In multivariate analysis, elevated AF levels of pentraxin 3 (*P* = 0.007) and resistin (*P* = 0.006), but not those of RAGE (*P* = 0.069), were independently associated with the occurrence of SPTD at <34 weeks after cerclage, following adjustment for baseline clinical variables (e.g., cervical dilation). The area under the curve (AUC) values of AF pentraxin 3, RAGE, and resistin for the prediction of SPTD at <34 weeks were 0.749, 0.669, and 0.770, respectively, which were similar to those of AF IL-6. However, in univariate analyses, no differences in the AF levels of endoglin, endostatin, haptoglobin, IGFBP-3, IGFBP-4, kallistatin, lumican, p-selectin, TGFBI, and VDBP were found to be associated with SPTD at <34 weeks after cerclage placement.

**Conclusions:**

In women with acute CI, the AF levels of pentraxin 3, RAGE, and resistin could be useful novel biomarkers for predicting SPTD following emergency cerclage. However, the clinical utility of these new biomarkers should be validated in larger multicenter studies.

## Introduction

Acute cervical insufficiency (CI), which has been characterized as an important cause of spontaneous preterm deliveries (SPTD) [1], complicates 0.5–1% of all pregnancies and is responsible for 5%–15% of pregnancy losses in the second trimester or extreme cases of SPTD (< 28 weeks) [1, 2]. In the context of acute CI, an emergency cerclage is the only accepted useful method for prolonging pregnancy and rescuing a fetus from abortion, resulting in neonatal survival rates of up to 72% [1, 3]. However, despite the relevance of this procedure in the treatment of acute CI, predictors of success in women undergoing emergency cerclage, except for inflammatory markers, remain understudied.

Acute CI is frequently associated with subclinical microbial invasion of the amniotic cavity (MIAC)/intra-amniotic inflammation (IAI), which is observed in more than half of such cases [4–7]. In particular, an emergency cervical cerclage has not been traditionally considered helpful in women with acute CI who were exposed to MIAC/IAI, since such patients demonstrate spontaneous preterm labor (PTL), premature ruptured membranes, or clinical chorioamnionitis [5, 7–10]. Hence, cervical cerclage is not recommended for such patients by some investigators [5, 8, 9]. However, a recent study by Oh et al. showed that MIAC/IAI in acute CI is treatable with antibiotic medications (ceftriaxone, clarithromycin, and metronidazole) that allow patients with CI and MIAC/IAI to deliver in ≥34 weeks [11]. These findings suggest that analysis of amniotic fluid (AF) via amniocentesis for identifying the presence of MIAC/IAI may be of clinical importance in patients with acute CI, for the decision-making process with respect to the management of the disease (cerclage and the use and type of antibiotics).

Previous reports have characterized several pro-inflammatory cytokines and chemokines and identified potential biomarkers in AF associated with poor pregnancy outcomes following an emergency cerclage [12–17]. However, clinical application of these markers remains limited owing to the lack of determination of diagnostic indices attributed to small sample sizes. Moreover, selected inflammatory markers in AF (e.g., pentraxin 3 (PTX3), the receptor for advanced glycation end products (RAGE), and resistin) that are involved in the mechanisms of preterm parturition in the context of PTL and preterm premature rupture of the membranes (PPROM) have not yet been fully studied in women with acute CI undergoing emergency cerclage [18–21]. To date, very few studies have reported the role of angiogenesis- and extracellular matrix-related proteins as markers for the determination of pregnancy outcomes after emergency cerclage, despite the fact that (1) angiogenesis and inflammation are mutually dependent [22, 23] and (2) matrix metalloproteinase (MMPs) may play an important role in the processes of premature cervical remodeling/ripening, which may contribute to cervical insufficiency [24, 25]. We sought to determine whether various novel inflammatory, angiogenic, and extracellular matrix-related mediators in AF can independently predict the emergency cerclage outcomes in women with acute CI.

## Materials and methods

### Study design and participants

A retrospective cohort study was conducted among 50 women with a singleton pregnancy at 18 to 25 weeks of gestation who underwent emergency cerclage following the diagnosis of acute CI and amniocentesis prior to cerclage placement. All pregnant women were recruited at the Seoul National University Bundang Hospital (Seongnamsi, Korea) between September 2004 and May 2018. AF samples of the participants were selected from a comprehensive database of patients who were admitted to the high-risk pregnancy unit at our institution if they met the following inclusion criteria: (i) singleton gestation with a live fetus; (ii) intact amniotic membranes visible through the cervical os confirmed by speculum examination; and (iii) availability of the aliquot of AF specimen for enzyme-linked immunosorbent assay (ELISA). The patients were excluded if they had (ⅰ) prophylactic cerclage during early pregnancy; (ⅱ) preterm labor syndrome or rupture of membranes at the diagnosis; (ⅲ) multiple pregnancies; (ⅳ) clinical chorioamnionitis at presentation; and (ⅴ) major congenital anomalies. A subset of participants in the present study was included in the previous studies, which showed the value of biomarkers for cerclage failure prediction using microarray and proteomic analyses [6, 13, 15]. A clinical diagnosis of acute CI was made when the intact fetal membranes were visible at the level of the external os or when they protruded into the vagina, as confirmed by sterile speculum examination with mid-trimester painless cervical dilation (≥ 1 cm). The primary outcome was SPTD at <34 weeks of gestation after cerclage placement, and the secondary outcomes were SPTD at <28 weeks of gestation and interval from cerclage to delivery. This study was approved by the ethics committee of Seoul National University Bundang Hospital, Seongnamsi, Korea (reference number: B-1311/228-010). All study participants provided written informed consent for the use of their stored biological specimens, as well as clinical data for research purposes. The study was conducted in accordance with the principles of the Declaration of Helsinki.

### Collection and processing of AF samples

A detailed description of AF sample collection and the detection of microorganisms has been provided in previous studies [26, 27]. Briefly, at the time of admission, but prior to cerclage placement, ultrasound-guided transabdominal amniocentesis was performed under aseptic conditions to assess the presence of MIAC/IAI and/or to reduce AF volume, for which the prolapsed fetal membranes were pushed back into the uterine cavity. The AF samples were cultured to identify the presence of microbes, such as aerobic/anaerobic bacteria, genital mycoplasmas (e.g., *Ureaplasma urealyticum* and *Mycoplasma hominis*), and fungi, as previously described [26]. The left-over AF samples were centrifuged for 10 min at 4°C, and the supernatant was aliquoted in 1.5 ml polypropylene tubes and stored at −70°C for further analysis.

### Assessment of AF mediators by ELISA

The stored AF samples were assayed using commercial ELISA kits for endoglin, endostatin, haptoglobin, IGFBP-3, -4, IL-6, kallistatin, lumican, macrophage colony-stimulating factor (M-CSF), PTX3, p-selectin, RAGE, resistin, transforming growth factor-beta-induced (TGFBI), and vitamin D-binding protein (VDBP) (DuoSet ELISA; R&D Systems, Minneapolis, MN, USA) according to the manufacturer’s instructions. These proteins were chosen for the study because they are important regulators of inflammation, angiogenesis, and extracellular matrix–related and immune responses (https://www.uniprot.org/); they are potential AF biomarkers for identifying patients with acute CI at high risk of SPTD after emergency cerclage based on our previous studies and other reports [13, 21, 28–34]; little information is currently available regarding their altered expression in AF associated with poor pregnancy outcomes after emergency cerclage; and an appropriate ELISA kit was commercially available. IL-6 levels were assayed in the AF for comparison with potential biomarkers assessed in this study, as it is a prototype marker for rescue cerclage failure [4, 6, 17, 35]. The AF dilutions used and the working range for each ELISA kit are described in detail in the Supplementary information. The intra-assay coefficient of variation (CV) was <10% for all analyzed proteins, except for IGFBP-3 (12.3%) and RAGE (13.3%). Inter-assay CV was not calculated in these ELISA experiments because all samples were analyzed in only one ELISA plate for each biomarker.

### Management of acute CI and clinical definitions

Decisions on the placement of an emergency cerclage and amniocentesis prior to procedure in acute CI pregnancies were made at the discretion of the attending physician. In our hospital, emergency cerclage placement for CI patients with overt chorioamnionitis, ruptured membranes, unexplained vaginal bleeding, and labor was contraindicated. During the study period, the McDonald cervical cerclage was performed as an emergency method by a maternal-fetal medicine attending physician under spinal anesthesia. All patients received postoperative antibiotics, but the administration of the type of antibiotic was at the discretion of the physician. Moreover, perioperative tocolytics and an intracervical Foley for the reduction of prolapsed fetal membranes were also used at the discretion of the obstetrician. Antenatal corticosteroids were administered to accelerate lung maturity between 23 and 34 weeks of gestation. A detailed description of the management of acute CI is provided in the Supplementary information. Clinical chorioamnionitis was diagnosed based on the criteria proposed by Gibbs et al [36]. IAI was defined with IL-6 concentrations of ≥ 2.6 ng/mL in the AF based on previous studies [11, 26].

### Statistical analysis

A two-tailed Mann-Whitney U test was used to compare continuous data between the study groups owing to small sample sizes. *χ*^2^-test or Fisher’s exact test was used as appropriate to compare categorical data between the two groups. Thereafter, multivariate Firth’s logistic regression analyses were performed to determine the independent relationship between various protein levels in the AF and the outcome measures, adjusting for the effect of baseline clinical covariates (such as cervical dilatation), which demonstrated a *P* value < 0.1 in univariate analysis. In the multivariate Firth’s logistic regression analyses, continuous independent variables were transformed into binary variables defined by cutoff values, according to their ROC curves, which were to be used for risk prediction and decision-making and to overcome the analytical limitations attributed to data distribution that was skewed to the left [37]. Receiver operating characteristic (ROC) curves were computed for each protein and used to determine the optimal cut-off values that would be predictive of SPTD at <34 and <28 weeks after cerclage placement. Optimal cut-offs were identified by maximizing Youden’s index (sensitivity + specificity − 1). The sensitivity, specificity, and accuracy of each protein, which were significantly associated with the outcome measures in univariate or multivariate models, were then calculated from the aforementioned optimal cut-offs. The areas under the ROC curves (AUCs) for each protein were calculated and compared using the DeLong method [38]. A Kaplan-Meier survival curve was constructed for low and high levels of each protein to analyze the interval from cerclage to delivery. The log-rank tests were used to examine if the two survival curves were different between the groups, and the statistically significant variables from the log-rank test were further characterized using Cox proportional hazards models for multivariate analysis. Statistical significance was defined as a two-tailed *P* value < 0.05. Statistical analyses of the data were carried out using SPSS version 25.0 (IBM SPSS Inc., Chicago, IL).

## Results

During the study period, 59 patients with acute CI underwent emergency cerclage; of them, 54 underwent amniocentesis and 5 did not. Among those 54 patients, no AF was available for assay for three and one was lost to follow-up, leaving 50 women in the final analysis. The mean gestational age (± standard deviation [SD]) of the study cohort was 21.6 ± 1.4 weeks (range, 18.3–25.4 weeks) at sampling and 31.1 ± 7.0 weeks (range, 21.2–40.5 weeks) at delivery, and the mean interval from cerclage to delivery was 65.7 ± 48.2 days. The prevalence of SPTDs at <34 and <28 weeks of gestation was 50.0% (25/50) and 40.0% (20/50), respectively. Three (6.0%, 3/50) patients had positive AF cultures, and 27 patients (54.0%, 27/50) had IAI.

### Demographic and clinical characteristics of the study population

The demographic and clinical characteristics of the study population are shown in Table 1. Patients who delivered at <34 weeks had significantly more advanced cervical dilatation at presentation and a higher rate of corticosteroid administration than those who delivered at ≥34 weeks. Univariate analyses conducted using SPTD at <28 weeks as the endpoint also revealed that the median cervical dilatation was higher in patients who delivered at <28 weeks than in those who delivered later on.

**Table 1:** Demographic and clinical characteristics of the study population.

Characteristics	Delivery < 34 weeks (n = 25)	Delivery ≥ 34 weeks (n = 25)	*P* values	Delivery < 28 weeks (n = 20)	Delivery ≥ 28 weeks (n = 30)	*P* values
Age (years)	31.48 ± 2.77	32.36 ± 4.22	0.347	31.85 ± 2.91	31.97 ± 3.99	0.960
Nulliparity	64.0% (16)	40.0% (10)	0.089	65.0% (13)	43.3% (13)	0.133
Gestational age at sampling (weeks)	21.56 ± 1.44	21.73 ± 1.49	0.861	21.40 ± 1.54	21.81 ± 1.39	0.506
Cervical dilatation (cm)	2.92 ±1.34	2.10 ± 1.61	**0.011**	3.00 ± 1.40	2.18 ± 1.54	**0.022**
≥ 3 cm	60.0% (15)	20.0% (5)	**0.004**	60.0% (12)	26.7% (8)	**0.018**
< 3 cm	40.0% (10)	80.0% (20)		40.0% (8)	73.3% (22)	
Use of tocolytics	64.0% (16)	44.0% (11)	0.156	65.0% (13)	46.7% (14)	0.203
Use of corticosteroids	48.0% (12)	12.0% (3)	**0.012**	40.0% (8)	23.3% (7)	0.208
Use of antibiotics	100.0% (25)	100.0% (25)		100.0% (20)	100.0% (30)	
Clinical chorioamnionitis	12.0% (3)	0% (0)	0.235	10.0% (2)	3.3% (1)	0.556
Gestational age at delivery (weeks)	24.7 ± 3.6	37.5 ± 1.6	**<0.001**	23.2 ± 1.6	36.4 ± 2.9	**<0.001**

Values are shown as the mean ± standard deviation or % (n).

### Various AF proteins in relation to SPTDs at <34 and <28 weeks of gestation

The median AF levels of IL-6, PTX3, RAGE, and resistin were significantly higher in patients who delivered at <34 weeks than in those who delivered at ≥34 weeks (Table 2). Univariate analysis with regard to SPTD at <28 vs. ≥28 weeks also showed that the median AF levels of IL-6, PTX3, and resistin were significantly higher in patients who delivered before 28 weeks than in those who delivered later on (Table 2). Moreover, M-CSF levels in the AF had a borderline association with SPTDs at <34 and <28 weeks after cerclage placement (*P* = 0.086 and *P* = 0.075, respectively), as determined by univariate analyses. However, in univariate analyses, no differences in the AF levels of endoglin, endostatin, haptoglobin, IGFBP-3, IGFBP-4, kallistatin, lumican, p-selectin, TGFBI, and VDBP were found to be associated with SPTD at <34 or <28 weeks after cerclage placement.

**Table 2:** Amniotic fluid levels of various proteins stratified according to spontaneous preterm delivery (SPTD) at < 28 and < 34 weeks.

Characteristics	Delivery < 34 weeks (n = 25)	Delivery ≥ 34 weeks (n = 25)	*P* values	Delivery < 28 weeks (n = 20)	Delivery ≥ 28 weeks (n = 30)	*P* values
AF endoglin (ng/mL)	11.11 ± 2.84	10.48 ± 2.07	0.317	11.27 ± 2.91	10.48 ± 2.14	0.193
AF endostatin (ng/mL)	73.43 ± 18.25	75.50 ± 22.27	0.952	75.13 ± 19.75	73.98 ± 20.72	0.625
AF haptoglobin (μg/mL)	2.19 ± 4.27	2.16 ± 3.22	0.648	1.78 ± 4.41	2.44 ± 3.28	0.178
AF IGFBP-3 (ng/mL)	700.66 ± 383.86	700.17 ± 382.08	0.720	673.58 ± 373.22	718.31 ± 388.16	0.984
AF IGFBP-4 (ng/mL)	430.20 ± 294.53	348.64 ± 173.77	0.273	451.35 ± 325.19	348.14 ± 161.11	0.205
AF IL-6 (ng/mL)	4.39 ± 2.21	2.59 ± 2.26	**0.008**	4.66 ± 2.12	2.72 ± 2.27	**0.008**
AF kallistatin (ng/mL)	913.60 ± 416.12	783.35 ± 293.27	0.497	923.92 ± 459.89	798.69 ± 276.31	0.823
AF lumican (μg/mL)	9.85 ± 3.10	9.77 ± 2.32	0.823	9.85 ± 3.35	9.78 ± 2.25	0.722
AF M-CSF (ng/mL)	3.28 ± 1.94	2.39 ± 0.97	0.086	3.50 ± 2.11	2.40 ± 0.90	0.075
AF pentraxin 3 (ng/mL)	21.78 ± 31.92	4.44 ± 7.80	**0.003**	24.72 ± 34.89	5.37 ± 8.42	**0.005**
AF p-selectin (ng/mL)	0.90 ± 0.88	0.62 ± 0.42	0.318	0.99 ± 0.96	0.61 ± 0.39	0.259
AF RAGE (ng/mL)	2361.82 ± 2538.61	1270.13 ± 832.71	**0.041**	2518.65 ± 2819.21	1347.52 ± 803.83	0.172
AF resistin (ng/mL)	187.43 ± 239.08	64.13 ± 59.88	**0.002**	187.43 ± 239.08	64.13 ± 59.88	**0.002**
AF TGFBI (μg/mL)	8.82 ± 4.10	7.95 ± 3.36	0.458	8.82 ± 4.10	7.95 ± 3.36	0.458
AF VDBP (μg/mL)	39.93 ± 15.31	38.31 ± 10.82	0.764	40.21 ± 16.78	38.39 ± 10.29	0.569
Positive amniotic fluid cultures	12.0% (3)	0% (0)	0.235	15.0% (3)	0% (0)	0.058
Intra-amniotic inflammation^†^	68.0% (17)	40% (10)	**0.047**	75.0% (15)	40% (12)	**0.015**

AF, amniotic fluid; IGFBP, insulin-like growth factor-binding protein; IL, interleukin; M-CSF, macrophage colony-stimulating factor; RAGE, receptor for advanced glycation end products; TGFBI, transforming growth factor beta-induced; VDBP, vitamin D-binding protein.

Values are shown as the mean ± standard deviation.

^**†**^Intra-amniotic inflammation was defined as AF IL-6 ≥ 2.6 ng/mL.

Multivariate Firth’s logistic regression analyses were further conducted to determine the independent association of the different protein levels in the AF with SPTDs at <34 and <28 weeks after adjusting for baseline covariates. Regarding the prediction of SPTD at <34 weeks, the optimal cut-off values used for dichotomization are listed as follows: ≥2.23 ng/mL for AF M-CSF, ≥2.34 ng/mL for AF PTX3, ≥1.30 ug/mL for AF RAGE, ≥62.87 ng/mL for AF resistin, and ≥3.0 cm for cervical dilatation at presentation. Multivariate logistic regression analyses confirmed that high AF levels of M-CSF, PTX3, and resistin (but not RAGE) were significantly associated with SPTD at <34 weeks after adjusting for parity, advanced cervical dilatation (≥3 cm), and corticosteroid administration (Table 3). Similarly, in the multivariate analysis, with respect to the prediction of SPTD at <28 weeks, high AF levels of PTX3 (≥2.34 ng/mL) and resistin (≥62.87 ng/mL) but not those of M-CSF (≥2.23 ng/mL) were still significantly independently associated with SPTD at <28 weeks when adjusting for advanced cervical dilatation (≥3.0 cm) (Table 3).

**Table 3:** Multivariate Firth’s logistic regression model showing the adjusted odds ratios of the association between the various amniotic fluid proteins and spontaneous preterm delivery (SPTD) after cerclage placement.

Variables^§^	SPTD at <34 weeks		SPTD at <28 weeks	
	Adjusted^†^	*P*-value*	Adjusted^‡^	*P*-value**
High AF M-CSF (≥2.23 ng/mL)	4.91 (1.04–33.22)	**0.045**	2.06 (0.60–7.29)	0.247
High AF pentraxin 3 (≥2.34 ng/mL)	8.02 (1.74–56.51)	**0.007**	4.71 (1.34–18.88)	**0.016**
High AF RAGE (≥1.30 ug/mL)	4.06 (0.90–24.68)	0.069		
High AF resistin (62.87 ng/mL)	7.78 (1.77–48.13)	**0.006**	5.68 (1.63–22.78)	**0.006**

AF, amniotic fluid; M-CSF, macrophage colony-stimulating factor; RAGE, receptor for advanced glycation end products

^†^ Adjustment for cervical dilatation (≥ 3 cm), use of corticosteroids, and parity.

* Adjusted odds ratio

^‡^ Adjusted for cervical dilatation (≥ 3 cm).

** For the adjusted odds ratio.

**Fig 1:**
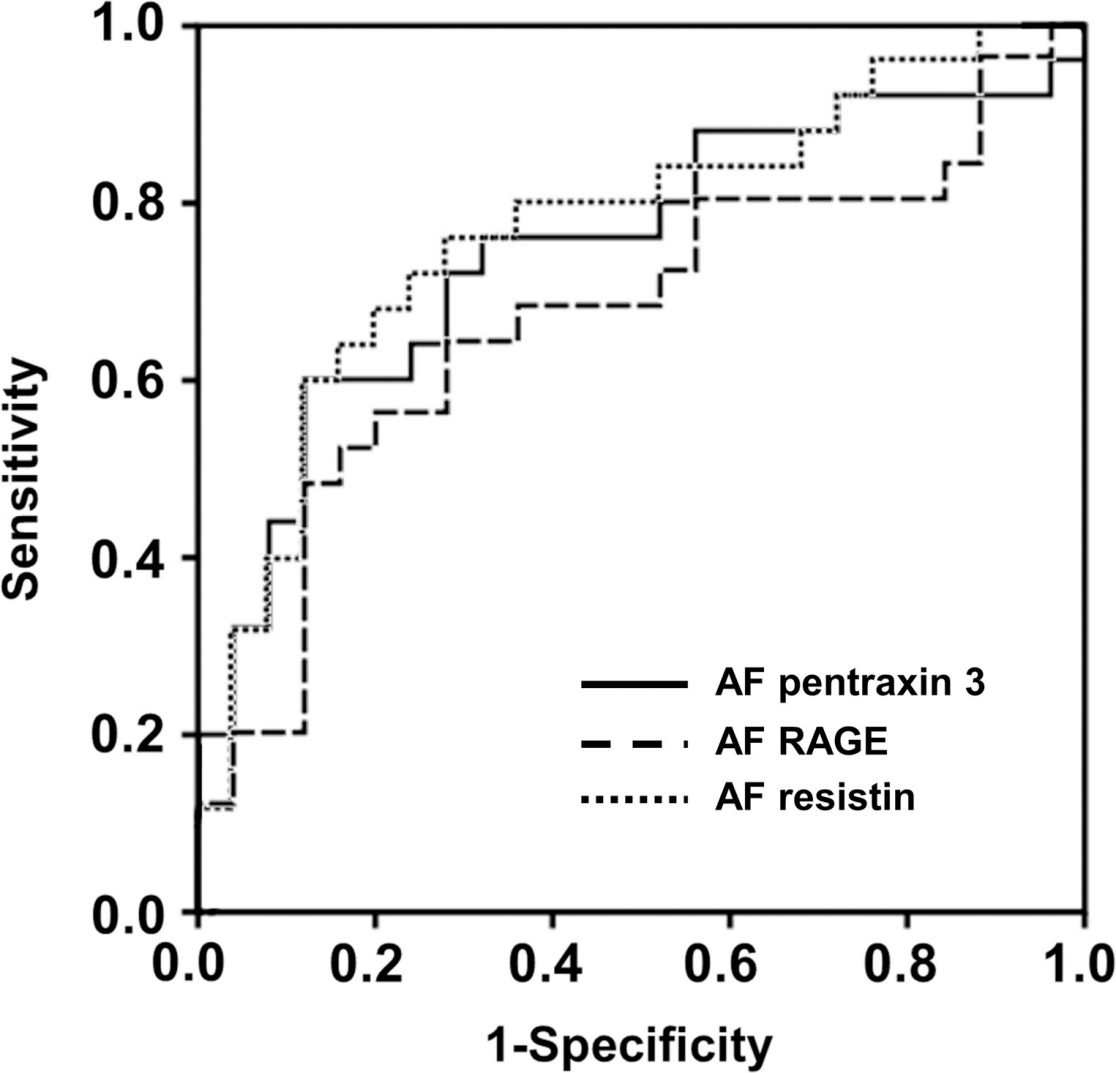
Receiver-operating characteristic curves for predicting spontaneous preterm delivery at <34 weeks of gestations by the pentraxin 3, RAGE, and resistin levels in AF. PTX3: AUC, 0.749 ± 0.071; *P* = 0.003. RAGE: AUC, 0.669 ± 0.079; *P* = 0.041. resistin: AUC, 0.770 ± 0.068; *P* = 0.001. AF, amniotic fluid; AUC, area under the curve ± standard error; RAGE, receptor for advanced glycation endproducts.

**Fig 2:**
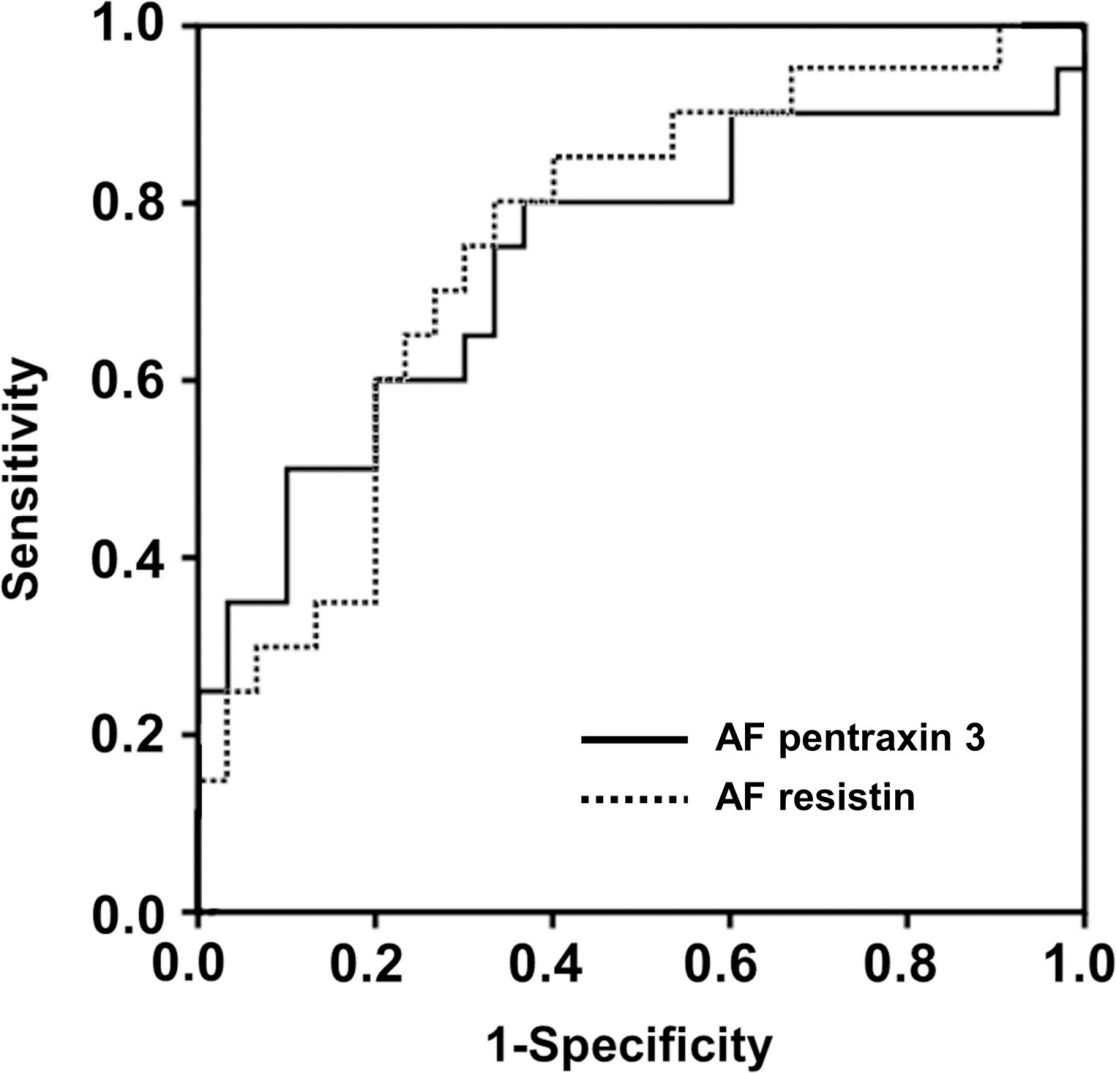
Receiver-operating characteristic curves for prediction of spontaneous preterm delivery at <28 weeks of gestations by the AF levels of the pentraxin 3 and resistin. PTX3: AUC, 0.737 ± 0.077; *P* = 0.005. resistin: AUC, 0.755 ± 0.070; *P* = 0.001. AF, amniotic fluid; AUC, area under the curve ± standard error.

Table 4 presents the diagnostic performances of various AF proteins with respect to the prediction of the occurrence of SPTDs at <34 and <28 weeks of gestation. For the prediction of the occurrence of SPTD at <34, the AUC values of AF PTX3, RAGE, and resistin were 0.749, 0.669, and 0.770, respectively (Table 4 and Fig 1), with no significant differences among them (all variables: *P* = 0.31–0.58), and no significant difference was observed compared to that in AF IL-6 (*P* = 0.30–0.68). Similarly, the AUC values for the prediction of SPTD at <28 weeks were 0.737 for AF PTX3 and 0.755 for AF resistin (Table 4 and Fig 2). The values were not significantly different between these two proteins (*P* = 0.66), as well as between these two proteins and IL-6 levels in the AF (*P* = 0.65 for PTX3 and *P* = 0.44 for resistin).

**Table 4:** Diagnostic indices of various amniotic fluid proteins and clinical factors to predict spontaneous preterm delivery (SPTD) after cerclage placement.

Variables	Area (± SE) under the ROC curve	95% CI	Cut-off value^†^	Sensitivity^‡^ (95% CI)	Specificity^‡^ (95% CI)	PPV	NPV
**SPTD at <34 weeks**							
AF pentraxin 3 (ng/mL)	0.749 ± 0.071	0.609–0.888	≥2.34	76.0 (54.9–90.6)	68.0 (46.5–85.1)	70.4	73.9
AF resistin (ng/mL)	0.770 ± 0.068	0.636–0.903	≥ 62.87	76.0 (54.9–90.6)	72.0 (50.6–87.9)	73.1	75.0
AF RAGE (ug/mL)	0.669 ± 0.079	0.514–0.823	≥ 1.30	64.0 (42.5–82.0)	72.0 (50.6–87.9)	69.6	66.7
AF IL-6 (ng/mL)	0.712 ± 0.074	0.567–0.857	≥4.21	68.0 (46.5–85.1)	76.0 (54.9–90.6)	73.9	70.4
AF M-CSF (ng/mL)	0.642 ± 0.079	0.487–0.797	≥2.23	68.0 (46.5–85.1)	60.0 (38.7–78.9)	62.9	65.2
Cervical dilatation (cm)	0.705 ± 0.075	0.557–0.852	≥3.0	60.0 (38.7–78.9)	80.0 (59.3–93.2)	75.0	66.7
**SPTD at <28 weeks**							
AF pentraxin 3 (ng/mL)	0.737 ± 0.077	0.586–0.888	≥2.34	80.0 (56.3–94.3)	63.3 (43.9–80.1)	59.3	82.6
AF resistin (ng/mL)	0.755 ± 0.070	0.617–0.893	≥ 62.87	80.0 (56.3–94.3)	66.7 (47.2–82.7)	61.5	83.3
AF IL-6 (ng/mL)	0.715 ± 0.075	0.569–0.861	≥4.21	75.0 (50.9–91.3)	73.3 (54.1–87.7)	65.2	81.5
Cervical dilatation (cm)	0.688 ± 0.077	0.538–0.839	≥3.0	60.0 (36.0–80.9)	73.3 (54.1–87.7)	60.0	73.3

SE, standard error; ROC, receiver operating characteristics; CI, confidence interval; PPV, positive predictive value; NPV, negative predictive value; AF, amniotic fluid; RAGE, receptor for advanced glycation end products; IL, interleukin; M-CSF, macrophage colony-stimulating factor.

^†^ Cut-off values corresponding to the highest sum of sensitivity and specificity

^‡^ Values are presented as % (95% CI).

### Association between AF proteins and interval from cerclage to delivery

**Fig 3:**
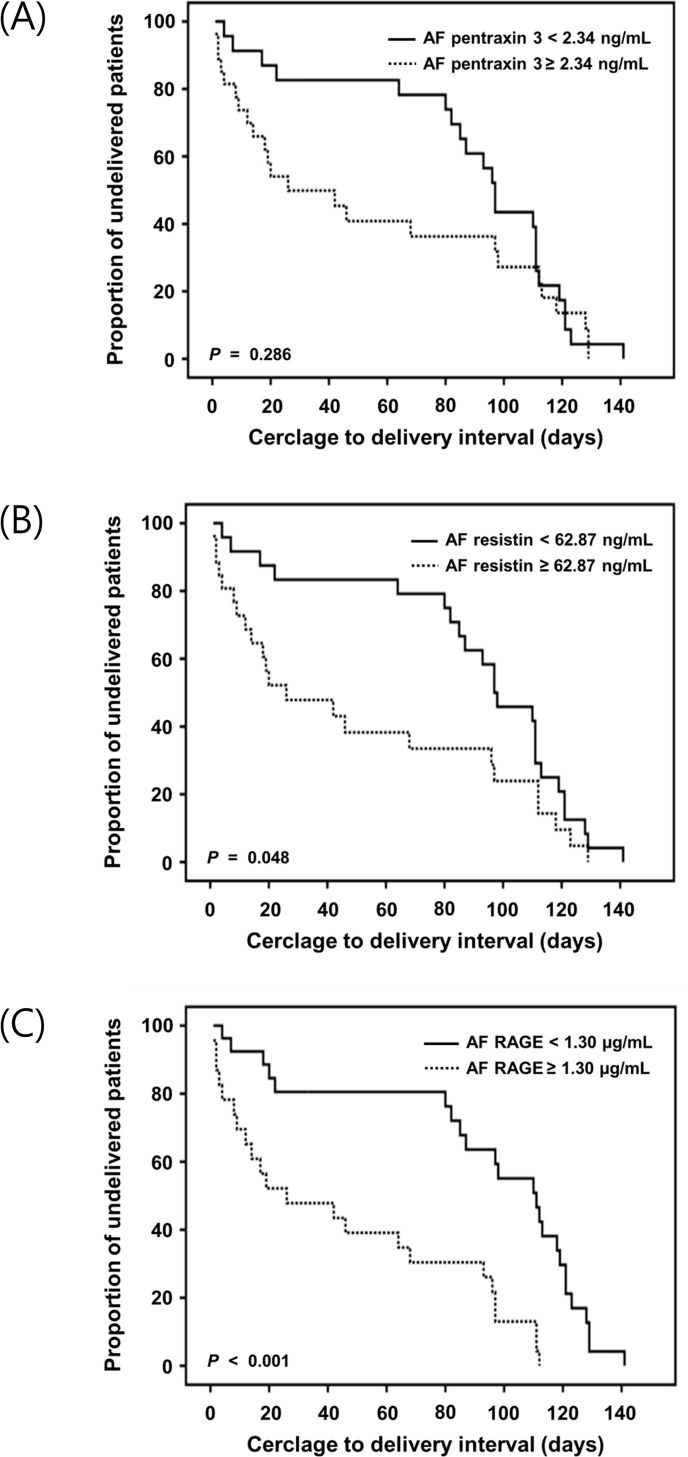
Kaplan-Meier survival estimates of the cerclage-to-delivery interval for (A) AF pentraxin 3 of ≥2.34 or <2.34 ng/mL (median, 26.00 days [95% CI, 0.00–56.13] vs. 97.00 days [95% CI, 90.79–103.21]; *P* = 0.286), (B) AF resistin of ≥ 62.87 or < 62.87 ng/mL (median, 26.00 days [95% CI, 0.00–59.77] vs. 97.00 days [95% CI, 76.59–117.40]; *P* = 0.048), and (C) AF RAGE of ≥1.30 or <1.30 μg/mL (median, 26.00 days [95% CI, 0.00–65.13] vs. 111.00 days [95% CI, 89.02–132.98]; *P* < 0.001). AF, amniotic fluid; CI, confidence interval; RAGE, receptor for advanced glycation endproducts.

Kaplan-Meier survival analyses performed using the cut-off values derived from ROC showed that patients with CI demonstrating high levels of RAGE in the AF (≥1.30 μg/mL; log-rank test, *P* < 0.001) or resistin (≥ 62.87 ng/mL; log-rank test, *P* = 0.048) undergoing emergency cerclage displayed significantly shorter intervals from cerclage to delivery (Fig 3). However, Kaplan-Meier and log-rank test analyses of the cerclage-to-delivery interval for a PTX3 of ≥2.34 or <2.34 ng/mL showed no statistically significant difference (log-rank test, *P* = 0.286). The Cox proportional hazards model indicated that high AF levels of RAGE but not those of PTX3 or resistin were significantly related to shorter cerclage-to-delivery intervals after controlling for parity, use of corticosteroids, and advanced cervical dilatation (≥ 3 cm) (Table 5).

**Table 5:** Cox proportional hazard analysis of cerclage-to-delivery interval.

Variable^†^	Adjusted hazard ratio ^‡^	95% confidence interval ^‡^	*P*-value *
High AF pentraxin 3 (≥2.34 ng/mL)	1.25	0.64–2.45	0.522
High AF RAGE (≥1.30 ug/mL)	4.09	1.89–8.89	<0.001
High AF resistin (62.87 ng/mL)	1.47	0.80–2.71	0.212

AF, amniotic fluid; RAGE, receptor for advanced glycation endproducts.

^†^ Variables were dichotomized as follows: high pentraxin 3 (≥2.34 ng/mL vs. <2.34 ng/mL), high RAGE (≥1.30 ug/mL vs. <1.30 ug/mL), and high resistin (≥ 62.87 ng/mL vs. < 62.87 ng/mL).

^‡^ Adjusted for cervical dilatation (≥ 3 cm), use of corticosteroids, and parity.

* Adjusted hazard ratio.

### Characteristics of three cases of delivery within 48 hours after cerclage

Table 6 presents the clinical and laboratory characteristics and outcomes of three patients who delivered within 48 hours after emergency cerclage. All patients in this group had IAI as well as elevated AF levels of IL-6, pentraxin 3, RAGE, and resistin, while one had a positive AF culture. The indications for delivery were progression of spontaneous labor with intact membranes (cases 1 and 2) and clinical chorioamnionitis after rupture of the membranes (case 3). A comparison of women with versus without SPTD within 48 hours after cerclage revealed no difference in AF levels of any proteins investigated in this study (S1 Table), although its statistical power was limited owing to the small number of cases of SPTD within 48 hours after cerclage (n = 3).

**Table 6:** Characteristics and outcomes of three patients who delivered within 48 hours after emergency cerclage.

	Gestational age (weeks)			Analysis of amniotic fluid						Treatment	
Case no.	Sampling	Delivery	Cervix dilatation (cm)	Culture	IAI^†^	IL-6 (ng/mL)	PTX3 (ng/mL)	RAGE (ug/mL)	Resistin (ng/mL)	Antibiotics/corticosteroid	Outcome
1	21.5	21.6	4.0	Neg	Yes	4.6	3.2	1.87	108.2	+/-	Spontaneous preterm labor and delivery
2	22.4	22.6	3.0	Neg	Yes	23.4	4.9	2.21	88.8	+/-	Spontaneous preterm labor and delivery
3	22.2	22.4	3.0	Pos	Yes	60.0	140.0	3.23	1000.0	+/-	Rupture of membranes, clinical chorioamnionitis

IAI, intra-amniotic inflammation; IL, interleukin; PTX3, pentraxin 3; RAGE, receptor for advanced glycation end products; Neg, negative; Pos, positive.

^†^Intra-amniotic inflammation was defined as amniotic fluid IL-6 ≥ 2.6 ng/mL.

## Discussion

In this study, we identified several novel AF biomarkers (such as PTX3, resistin, and RAGE) that can identify patients with acute CI at high risk of early and late SPTD after emergency cerclage. All markers were associated with inflammation and the first two (i.e., PTX3 and resistin), in particular, were independent of cervical dilatation. However, the angiogenic (endoglin, endostatin, and IGFBP-3, and -4), extracellular matrix- (lumican, TGFBI), and other inflammation-related proteins (haptoglobin, kallistatin, M-CSF, p-selectin, and VDBP) were not significantly associated with poor clinical outcomes for emergency cerclage performed for the treatment of acute CI. These data provide insight into therapeutic targets for prolonging gestational latency in acute CI and their role in the development of SPTD after emergency cerclage.

IAI and/or MIAC, as well as advanced cervical dilation at presentation, have been consistently reported as the most important risk factors for poor pregnancy outcomes of emergency cerclage for CI [4–6, 14, 39–42]. In particular, sterile IAI is a common phenotype of IAI observed in pregnancies with CI with protruding fetal membranes [4, 7]. It is found in over 50% of these pregnancies and is associated with worse outcomes in this context [4, 7, 35, 43]. These findings are in line with the data derived from the present study. From a clinical perspective, it is important to determine whether amniocentesis is necessary for the management of acute CI and whether emergency cerclage is a feasible and beneficial treatment option for patients with acute CI diagnosed with MIAC/IAI. To the best of our knowledge, some clinicians may be reluctant to perform emergency cerclage for acute CI in the context of MIAC/IAI, because they assume that the inflammatory response already induced in the amniotic cavity likely puts such patients at a higher risk of early SPTD, regardless of suitable interventions (i.e., emergency cerclage and antibiotic therapy). However, recent studies on patients with acute CI with prolapsed fetal membranes complicated by MIAC/IAI revealed that antibiotic therapy (especially clarithromycin for mycoplasmas) may reduce or even resolve IAI in a fraction of cases, especially in those with milder forms of sterile IAI [4, 11]. Moreover, a previous randomized controlled trial conducted among patients with acute CI undergoing an examination-indicated cerclage demonstrated that a higher proportion of pregnancies was prolonged by at least 28 days among women who received perioperative indomethacin and antibiotics [44]. Taken together, these data may support the application of amniocentesis in the setting of emergency cerclage placement for acute CI to select the patients who benefited from antibiotics and potential therapeutic inhibitors of inflammation. A case-by-case selection of individuals with CI diagnosed with MIAC/IAI that would benefit from cerclage is certainly appropriate; however, we believe that emergency cerclage might be considered for such patients in the absence of labor or clinical chorioamnionitis. Further larger prospective multicenter randomized studies are required to compare the effectiveness of emergency cerclage placement versus expectant management in a subset of patients with acute CI with MIAC/IAI.

Notably, the present study shows for the first time that PTX3, resistin, and RAGE in AF are novel markers for predicting early and late SPTD following the placement of emergency cerclage for CI. In particular, these markers, with moderate diagnostic performance, revealed diagnostic accuracies similar to those of AF IL-6, which could be considered as a prototype AF-based test factor. Similar results have been documented in the context of PTL where altered expression of the aforementioned proteins in the AF was significantly associated with SPTD and MIAC/IAI [45–49]. PTX3, a prototype of the long pentraxin family, is an acute-phase response protein that is significantly involved in the regulation of innate immunity and inflammation [50, 51]. This protein is produced in local inflammatory lesions by various cell types, including macrophages, monocytes, vascular smooth muscle cells, and endothelial cells [50]. In line with the known biological role and site of production of PTX3, previous studies on AF derived from women with PPROM/PTL showed that elevated AF concentrations of PTX3 are associated with IAI and histological chorioamnionitis [18, 19, 52, 53]. However, to date, no data are available on whether the changes in PTX3 concentrations in AF are associated with poor pregnancy outcomes following emergency cerclage for CI. Herein, AF PTX3 was found to be independently related to poor prognosis after emergency cerclage, which is expected considering the pivotal involvement of AF PTX3 in the pathogenesis of IAI and the importance of IAI in association with poor clinical prognosis in the setting of emergency cerclage placement [4, 19, 42, 52, 53].

Human resistin [also known as adipose tissue-specific secretory factor (ADSF)] is a 12.5 kDa cysteine-rich peptide that is primarily produced by macrophages and monocytes (although in rodents, its main source is white adipose tissue) [54, 55]. In humans, this protein plays an important role in inflammatory responses throughout the body, and resistin itself, as a pro-inflammatory cytokine, can activate immune cells and promote the release of several pro-inflammatory cytokines [55]. In the setting of PPROM and PTL, previous research on AF has demonstrated that increased levels of resistin are associated with IAI, histological chorioamnionitis, and SPTD in the absence of IAI [20, 56], which is consistent with the pivotal role of resistin in inflammation and inflammation-associated diseases (e.g., arthritis and atherosclerosis) [54, 57], as well as in general with the findings reported herein for AF resistin.

RAGE is a transmembrane multi-ligand receptor that belongs to the immunoglobulin superfamily and is expressed in the amnion epithelial, decidual, and extravillous trophoblastic cells in the fetal membranes and placenta [58, 59]. This protein binds to several ligands, including EN-RAGE (calgranulins) and high-mobility group box 1 (HMGB1), and activation of RAGE by its ligands is involved in the inflammatory response mediated via the induction of nuclear factor kappaB signaling, leading to the production of pro-inflammatory cytokines [58, 60]. In addition to signaling, ligand-activated RAGE also functions as an adhesive receptor that interacts with integrins, thereby directly being involved in the recruitment of pro-inflammatory leukocytes to the inflammatory site [60, 61]. In previous studies, soluble RAGE (sRAGE) was found to be a physiologic constituent of AF, and its concentration increased with advancing gestational age [21, 59]. The observed significant increase in RAGE expression in AF derived from patients with SPTD following cerclage placement may reflect a response to the increase in inflammation/infection observed in the amniotic cavity and decidua, leading to the terminal cascade of events resulting in SPTD. Previously, Romero et al. showed that AF levels of sRAGE in PTL/PPROM were significantly elevated in women with MIAC/IAI [21]. The RAGE results demonstrated in the present study are consistent with this previous finding [21], along with the reported associations between IAI/MIAC and SPTD risk after emergency cerclage [4–6, 12, 14].

Unlike previous studies conducted in women with PTL, where SPTD and MIAC/IAI were closely associated with high expression of angiogenesis-related factors in AF, such as endoglin, IGFBP-3, and VEGFR-1 [30, 62, 63], we found that the changes in angiogenesis-related biomarker levels in AF were not associated with SPTD development after cerclage placement. This discrepancy regarding the role of angiogenesis-related factors in the regulation of SPTD between acute CI and PTL may be attributed to the sequential expression of inflammatory cytokines, angiogenesis-inducing factors, and matrix-degrading enzymes during local inflammation [64]. In AF samples obtained at the time of amniocentesis, IAI in patients with acute CI may be detected at early stages of inflammation in the amniotic cavity; thus, angiogenesis-related factors were not yet activated by inflammatory cytokines. However, IAI in the setting of PTL is likely to represent a more advanced stage of inflammation in AF to the extent that it may sufficiently induce contractions by locally produced prostaglandins; hence, angiogenesis-related factors were already upregulated in the AF, along with the upregulation of inflammatory factors [65, 66]. This discrepancy may be attributed to the differences in sample size between the studies.

In the context of CI (regardless of cerclage status), PTL, and PPROM, MIAC is reportedly associated with an increased risk of preterm delivery [7, 49, 67–70]. However, we cannot confirm this association in the current study of patients undergoing emergency cerclage for CI. This discrepancy between our data and those of other investigators can be explained as follows. First, the very small number of MIAC cases (n = 3) in this study may limit the statistical power of MIAC-related outcomes. Second, in the present study, MIAC was detected by conventional culture but not by polymerase chain reaction (PCR), thereby leading to false-negative MIAC results. Indeed, Combs et al. reported that the traditional culture- and PCR-based methods are complementary rather than substitutes for each other in the detection of MIAC [69].

The present study had several limitations. First, the study was retrospective in nature and was conducted in a single institution, and external validation of our findings (especially, the suggested cut-off values for analytes) was not performed in a different set of patients, all of which limit the generalizability of the results. Second, the present study included a relatively small number of study subjects because of the very low incidence of emergency cerclage for acute CI [2]. It may cause not only type II error (false-negative results for biomarkers assessed in the present study) but also lead to an overestimation of sensitivity and specificity [71]. Thus, the present data can be considered preliminary and should be validated in larger and different cohorts. Third, the measurements of AF-based biomarkers require invasive amniocentesis, which may limit routine clinical application in acute settings. Fourth, uniform treatment, especially antibiotic type, was not provided to all women with CI undergoing rescue cerclage because study subjects over a 13-year period were included to achieve a larger data set. In fact, *Ureaplasma* spp. is the most frequently isolated microorganisms in the AF from women with CI [7, 67, 70], while clarithromycin is more effective for the treatment of MIAC with *Ureaplasma* spp. than erythromycin or azithromycin [11, 72]. The present study had the following strengths: (1) this was the first study in which angiogenesis-, extracellular matrix-, and inflammation-associated mediators, which have not yet been explored in the context of acute CI, were evaluated in the AF from patients undergoing emergency cerclage; (2) adjustment was performed for known relevant confounding variables (e.g., cervical dilatation) in the context of emergency cerclage, which was examined with logistic regression; and (3) the study comprised homogeneous cohort of Korean patients with CI. In the present study, SPTD at <34 weeks following emergency cerclage was selected as the primary outcome measure because babies born at ≥34 weeks had much lower risks of mortality and morbidity [73, 74].

## Conclusions

In conclusion, our results suggest that AF levels of PTX3, resistin, and RAGE could act as novel potential biomarkers indicating the development of SPTD after emergency cerclage for acute CI. However, altered AF levels of angiogenesis- and extracellular matrix-related mediators are not associated with adverse clinical outcomes after emergency cerclage. These data highlight the importance of inflammatory response in the amniotic cavity as underlying molecular mechanisms involved in the development of SPTD after emergency cerclage and may be helpful to clinicians in estimating the effectiveness of cerclage placement and in counseling patients undergoing this procedure.

## Supporting information

S1 Table Amniotic fluid levels of various proteins stratified according to spontaneous preterm delivery (SPTD) within 48 hours after emergency cerclage. (DOCX)

S1 File Raw data for the total cohort. (SAV)

S2 File Detailed descriptions of analysis of various proteins in the amniotic fluid and management of cervical insufficiency. (DOCX)
