# Supplementary material for: Prediction of emergency cerclage outcomes in women with cervical insufficiency: The role of inflammatory, angiogenic, and extracellular matrix-related proteins in amniotic fluid
Source: PLoS One. 2022 May 10;17(5):e0268291. doi: 10.1371/journal.pone.0268291 (PMC9089878; doi:10.1371/journal.pone.0268291)
Supplement: S1 Table — (DOCX) [file pone.0268291.s001.docx]

## S1 Table Amniotic fluid levels of various proteins stratified according to spontaneous preterm delivery (SPTD) within 48 hours after emergency cerclage

| Characteristics | SPTD after emergency cerclage | | *P* values |
| --- | --- | --- | --- |
|  | Delivery ≤ 48 hours  (n =3) | Delivery > 48 hours  (n = 47) |  |
| AF endoglin (ng/mL) | 12.53 ± 1.94 | 10.69 ± 2.49 | 0.196 |
| AF endostatin (ng/mL) | 78.00 ± 18.49 | 74.22 ± 20.40 | 0.707 |
| AF haptoglobin (µg/mL) | 0.60 ± 0.35 | 2.28 ± 0.38 | 0.984 |
| AF IGFBP-3 (ng/mL) | 598.11 ± 481.37 | 706.94 ± 377.15 | 0.728 |
| AF IGFBP-4 (ng/mL) | 366.77 ± 196.10 | 390.87 ± 247.21 | 0.854 |
| AF IL-6 (ng/mL) | 29.32 ± 28.18 | 12.36 ± 17.40 | 0.107 |
| AF kallistatin (ng/mL) | 898.07 ± 611.78 | 846.66 ± 352.32 | 0.770 |
| AF lumican (µg/mL) | 8.55 ± 5.51 | 9.89 ± 2.53 | 0.951 |
| AF M-CSF (ng/mL) | 5.16 ± 3.29 | 2.69 ± 1.35 | 0.069 |
| AF pentraxin 3 (ng/mL) | 49.36 ± 78.50 | 10.80 ± 16.91 | 0.184 |
| AF p-selectin (ng/mL) | 0.67 ± 0.68 | 0.77 ± 0.70 | 0.581 |
| AF RAGE (ng/mL) | 2439.27 ± 710.46 | 1776.19 ± 1999.77 | 0.076 |
| AF resistin (ng/mL) | 399.01 ± 520.56 | 95.22 ± 111.34 | 0.098 |
| AF TGFBI (µg/mL) | 9.33 ± 5.92 | 8.23 ± 3.56 | 0.822 |
| AF VDBP (µg/mL) | 31.63 ± 9.22 | 39.63 ± 13.33 | 0.227 |
| Positive amniotic fluid cultures | 33.3 % (1/3) | 4.3 % (2/47) | 0.173 |
| Intra-amniotic inflammation^†^ | 100 % (3/3) | 51.1 % (24/47) | 0.240 |

AF, amniotic fluid; IGFBP, insulin-like growth factor-binding protein; IL, interleukin; M-CSF, macrophage colony-stimulating factor; RAGE, receptor for advanced glycation end products; TGFBI, transforming growth factor beta-induced; VDBP, vitamin D-binding protein.

## Values are shown as the mean ± standard deviation.

^†^Intra-amniotic inflammation was defined as AF IL-6 ≥ 2.6 ng/mL.
