# Supplementary material for: Prediction of emergency cerclage outcomes in women with cervical insufficiency: The role of inflammatory, angiogenic, and extracellular matrix-related proteins in amniotic fluid
Source: PLoS One. 2022 May 10;17(5):e0268291. doi: 10.1371/journal.pone.0268291 (PMC9089878; doi:10.1371/journal.pone.0268291)
Supplement: S2 File — (DOCX) [file pone.0268291.s003.docx]

## - Supplementary Materials -

**Analysis of various proteins in the amniotic fluid**

The ranges of endoglin, endostatin, haptoglobin, IGFBP-3, IGFBP-4, IL-6, kallistatin, lumican, M-CSF, pentraxin 3, p-selectin, RAGE, resistin, TGFBI, and VDBP standard curves were 125-8000 pg/mL, 62.5-4000 pg/mL, 31.2-2000 pg/mL, 125-8000 pg/mL, 0.5-32 ng/mL, 9.4-600 pg/mL, 125~8000 pg/mL, 125-8000pg/mL, 15.6-1000 pg/mL, 218-14000 pg/mL, 125-8000pg/mL, 62.5-4000 pg/mL, 31.2-2000pg/mL, 62.5-4000 pg/mL, and 187.5-3000 pg/mL, respectively. Prior to measurement of these proteins, the AF samples were diluted at 1:4 for p-selectin and RAGE, 1:10 for endoglin, IL-6, M-CSF, and pentraxin 3, 1:100 for endostatin, 1:200 for IGFBP-4, 1:500 for kallistatin and resistin, 1:1000 for IGFBP-3 and TGFBI, 1:5,000 for lumican, 1:10,000 for haptoglobin, and 1:50,000 for VDBP. The intra-assay coefficients of variation were 2.0% for endoglin, 1.2% for endostatin, 3.3% for haptoglobin, 12.3% for IGFBP-3, 3.6% for IGFBP-4, 2.9% for IL-6, 2.6% for kallistatin, 4.2% for lumican, 5.0% for M-CSF, 1.4% for pentraxin 3, 3.9% for p-selectin, 13.3% for RAGE, 5.3% for resistin, 5.0% for TGFBI, and 0.6% for VDBP, respectively.

Management of **c**ervical insufficiency

Emergency cerclage was offered to patients with cervical insufficiency and performed with the McDonald technique under spinal anesthesia. Prophylactic broad-spectrum antibiotics were administered in the operating room and after surgery in all women with cervical insufficiency. Tocolytics (magnesium sulfate, ritodrine or atosiban) were used at the discretion of the attending obstetrician when regular uterine contractions had developed. Patients who chose expectant management instead of cerclage were also given similar multifactorial treatments during their hospitalization, including prophylactic broad-spectrum antibiotics and tocolytic drugs at the discretion of their obstetricians. Decisions on the treatment for acute CI, such as the placement of a rescue cerclage and amniocentesis, were made at the discretion of attending obstetrician.

Clinical chorioamnionitis was diagnosed following the criteria proposed by Gibbs et al.^1^; fever (≥37.8°C) and the presence of two or more of the associated clinical findings (uterine tenderness, malodorous vaginal discharge, maternal leukocytosis, maternal tachycardia, and fetal tachycardia).

**Supplementary References**

1. Gibbs RS, Blanco JD, St Clair PJ, Castaneda YS. Quantitative bacteriology of amniotic fluid from women with clinical intraamniotic infection at term. The Journal of infectious diseases 1982; 145(1): 1-8.
